# Baseline CXCL10 and CXCL13 levels are predictive biomarkers for tumor necrosis factor inhibitor therapy in patients with moderate to severe rheumatoid arthritis: a pilot, prospective study

**DOI:** 10.1186/s13075-016-0995-0

**Published:** 2016-04-22

**Authors:** Bobby Kwanghoon Han, Igor Kuzin, John P. Gaughan, Nancy J. Olsen, Andrea Bottaro

**Affiliations:** Division of Rheumatology, Cooper Medical School of Rowan University, Camden, NJ 08103 USA; Department of Biomedical Sciences, Cooper Medical School of Rowan University, Camden, NJ 08103 USA; Cooper Research Institute, Cooper Medical School of Rowan University, Camden, NJ 08103 USA; Division of Rheumatology, Department of Medicine, Penn State MS Hershey Medical Center, Hershey, PA 17033 USA

**Keywords:** CXCL10, CXCL13, TNF inhibitor, Rheumatoid arthritis

## Abstract

**Background:**

TNF inhibitors have been used as a treatment for moderate to severe RA patients. However, reliable biomarkers that predict therapeutic response to TNF inhibitors are lacking. In this study, we investigated whether chemokines may represent useful biomarkers to predict the response to TNF inhibitor therapy in RA.

**Methods:**

RA patients (n = 29) who were initiating adalimumab or etanercept were recruited from the rheumatology clinics at Cooper University Hospital. RA patients were evaluated at baseline and 14 weeks after TNF inhibitor therapy, and serum levels of CXCL10, CXCL13, and CCL20 were measured by ELISA. Responders (n = 16) were defined as patients who had good or moderate response at week 14 by EULAR response criteria, and nonresponders (n = 13) were defined as having no response.

**Results:**

Responders had higher levels of baseline CXCL10 and CXCL13 compared to nonresponders (*p* = 0.03 and 0.002 respectively). There was no difference in CCL20 levels. CXCL10 and CXCL13 were highly correlated with each other, and were higher in seropositive RA patients. CXCL10 and CXCL13 levels were decreased after TNF inhibitor therapy in responders. Baseline additive levels of CXCL10 + 13 were correlated with changes in DAS score at 14 weeks after TNF inhibitor therapy (r = 0.42, *p* = 0.03), and ROC curve analyses for predictive ability of CXCL10 + 13 showed an AUC of 0.83.

**Conclusions:**

Elevated baseline levels of CXCL10 and CXCL13 were associated with favorable response to TNF inhibitor therapy in RA. Subjects with high CXCL10 and high CXCL13 may represent a subset of RA patients whose inflammatory reactions are primarily driven by TNF.

## Background

Rheumatoid arthritis (RA) is a disease that is characterized by synovial inflammation, cartilage and bone destruction, and systemic features [1, 2]. Advances in understanding the pathogenesis of the disease have fostered the development of new therapeutics. Tumor necrosis factor (TNF) inhibitors are used as a treatment for moderate to severe RA patients who have inadequate responses to conventional disease-modifying antirheumatic drugs (DMARDs) including methotrexate. However, reliable predictive biomarkers of therapeutic response for TNF inhibitor therapy are lacking [3].

Several studies have been conducted to discover predictive biomarkers for RA therapies. It has been reported that type I interferon (IFN) signature is associated with the therapeutic response to TNF inhibitors and rituximab [4–7]. C-X-C motif chemokine 10 (CXCL10) is induced by type I and II IFNs [8, 9]. Recruitment and activation of C-X-C chemokine receptor type 3 (CXCR3)-positive T helper 1 (Th1) lymphocytes and monocytes by CXCL10 may lead to TNF production in RA [10, 11]. In other studies, C-X-C motif chemokine 13 (CXCL13) has been reported to be a predictive biomarker for RA therapies [12, 13]. CXCL13, a chemokine that attracts B lymphocytes and follicular helper T lymphocytes (TFH) by binding to C-X-C chemokine receptor type 5 (CXCR5), is upregulated in a subset of T cells by TNF or interleukin 6 (IL-6) in RA [14, 15] as well as in follicular dendritic cells in the germinal center of lymphoid tissues [16]. Lastly, baseline interleukin 17 (IL-17) levels are inversely correlated with response to TNF inhibitor therapy in RA [17]. C-C motif chemokine 20 (CCL20) attracts T helper 17 (Th17) lymphocytes expressing C-C chemokine receptor type 6 (CCR6), and induced by interleukin 1β (IL-1β), IL-17, and TNF [18, 19].

In this study, we investigated whether these three chemokines may represent useful predictive biomarkers for TNF inhibitor therapy in RA patients. We measured CXCL10, CXCL13, and CCL20 in RA patients who were initiating TNF inhibitor therapy and correlated each chemokine level with the therapeutic response.

## Methods

### Patients and assessment

Patients with RA who met the inclusion and exclusion criteria were recruited during routine care in the rheumatology clinics at the Cooper University Hospital (Camden, NJ, USA). The inclusion criteria for this study were: (1) diagnosis of RA by American College of Rheumatology (ACR) criteria; (2) active RA defined by disease activity score (DAS) >4.4; (3) inadequate response to methotrexate; (4) clinical indication for initiating adalimumab or etanercept treatment. The exclusion criteria were: (1) diagnosis of other connective tissue diseases including systemic lupus erythematosus, systemic sclerosis, or dermatomyositis, or interstitial lung disease; (2) diagnosis of chronic infection including viral hepatitis or human immunodeficiency virus; (3) history of malignancy. All 29 patients except one were continued on baseline methotrexate and other DMARDs while taking adalimumab (22 patients) or etanercept (7 patients). The patients were assessed and peripheral blood samples were obtained at baseline and 14 weeks after TNF inhibitor therapy. The results of rheumatoid factor (RF), anti-cyclic citrullinated peptide antibody (anti-CCP), and erythrocyte sedimentation rate (ESR) tests were obtained as part of patient care. Responders were defined as patients who had good to moderate response at week 14 by European League Against Rheumatism (EULAR) response criteria (14 with adalimumab and 2 with etanercept treatment), and nonresponders were defined as having no response (8 with adalimumab and 5 with etanercept treatment). The research protocol was approved by the Institutional Review Board of Cooper University Hospital and all patients provided written informed consent for participation in the study.

### Measurement of chemokine levels

Freshly isolated serum samples were aliquoted and stored in a −80 °C freezer until use. Commercial enzyme-linked immunosorbent assay (ELISA) kits were used for serum measurements of CXCL10 (R&D, Minneapolis, MN, USA), CXCL13 (Sigma-Aldrich, St Louis, MO, USA), and CCL20 (Sigma-Aldrich, St Louis, MO, USA).

### Statistical analyses

Statistical analyses were performed using SAS v9.4 (SAS Institute, Cary, NC, USA), and graphs were generated using GraphPad Prism 6.0 (GraphPad Software, La Jolla, CA, USA). Continuous variables were compared using Wilcoxon ranked sum test, and dichotomous variables were compared using Fisher’s exact test. Correlations between pairs of continuous variables were performed using Spearman correlation coefficient. Differences between pretreatment and posttreatment chemokine levels were compared using analysis of variance (ANOVA) for repeated measures. Receiver operating characteristic (ROC) curve analysis was performed to assess the predictive ability of cytokine variables. In all the tests, a two-sided *p* value <0.05 was considered significant.

## Results

### Baseline serum CXCL10 and CXCL13 levels are higher in responders to TNF inhibitor therapy

Twenty-nine RA patients who were about to start either adalimumab or etanercept after having an inadequate response to methotrexate and other DMARDs were recruited. Five patients had been treated with a TNF inhibitor previously, and their last treatment was at least 3 months ago. After 14 weeks of TNF inhibitor therapy, using EULAR response criteria, the patients were classified into 16 good and moderate responders (collectively termed hereafter as ‘responders’) and 13 nonresponders. Their baseline characteristics, summarized in Table 1, showed no significant differences between responders and nonresponders.

**Table 1:** Baseline characteristics of RA patients

	Responders (n = 16)	Nonresponders (n = 13)	*p* value
Age (years)	51.6 ± 12.7	50.7 ± 8.1	0.80^a^
Gender (female %)	69 (11/16)	77 (10/13)	0.70^b^
Duration (years)	6.5 ± 4.9	7.4 ± 7.5	0.66^a^
RF or anti-CCP positive (%)	75 (12/16)	54 (7/13)	0.27^b^
DAS28 ESR	6.2 ± 1.1	6.7 ± 0.6	0.15^a^
ESR (mm)	37 ± 31	30 ± 22	0.79^a^

Values are presented as mean with standard deviation. *P* values were determined by Wilcoxon ranked sum test^a^ and by Fisher’s exact test^b^

*RF* rheumatoid factor, anti-CCP anti-cyclic citrullinated peptide​, *DAS28 ESR* disease activity score in 28 joints based on erythrocyte sedimentation rate, *ESR* erythrocyte sedimentation rate

**Fig. 1:**
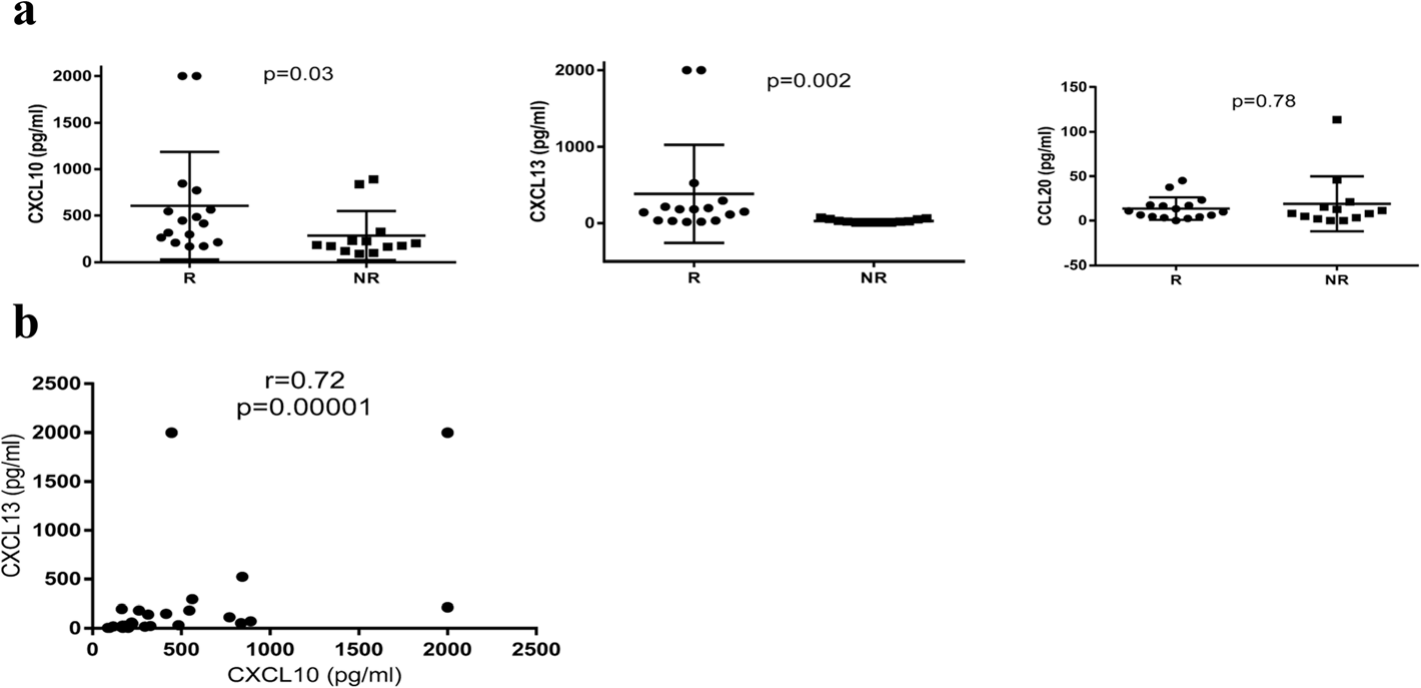
**a** Baseline serum chemokine levels in responders (n = 16) and nonresponders (n = 13) to TNF inhibitor therapy. CXCL10 (*p* = 0.03) and CXCL13 (*p* = 0.002) levels were higher in responders than in nonresponders. The chemokine levels in the two groups were compared using Wilcoxon ranked sum test. Bars represent mean ± standard deviation. *R* responders, *NR* nonresponders. **b** Correlation between baseline CXCL10 and CXCL13 levels. Baseline CXCL10 and CXCL13 levels were highly correlated (r = 0.72, *p* = 0.00001). The association between the two chemokine levels was assessed using Spearman correlation

Baseline chemokine levels were measured by ELISA before starting TNF inhibitor therapy and compared between responders and nonresponders (Fig. 1a). Responders had significantly higher serum levels of CXCL10 (606 ± 581 vs 283 ± 265 pg/ml, *p* = 0.03) and CXCL13 (383 ± 644 vs 27 ± 24 pg/ml, *p* = 0.002) compared to nonresponders. There was no difference in CCL20 levels between responders and nonresponders (14 ± 13 vs 19 ± 31 pg/ml, *p* = 0.78).

Among patients who were treated with adalimumab (n = 22), baseline CXCL10 and CXCL13 levels were higher in responders than in nonresponders (*p* = 0.02 and *p* = 0.04 respectively). Among patients who were treated with etanercept (n = 7), CXCL13 levels were higher in responders (*p* = 0.045), and CXCL10 levels were numerically higher in responders, but the difference did not reach statistical significance (*p* = 0.20).

### CXCL10 and CXCL13 levels are correlated with each other and are higher in seropositive RA patients

The relationship between chemokine levels and disease activity was assessed. Baseline or posttreatment levels of CXCL10, CXCL13, or CCL20 were not correlated with DAS28 or ESR (data not shown). Of note, baseline and posttreatment levels of CXCL10 and CXCL13 were correlated with each other (r = 0.72, *p* = 0.00001 and r = 0.59, *p* = 0.001 respectively) (Fig. 1b).

Chemokine levels were compared between seropositive and seronegative patients. Baseline CXCL10 and CXCL13 levels were higher in anti-CCP-positive patients than in anti-CCP-negative patients (*p* = 0.02 and *p* = 0.005 respectively). Only baseline CXCL13 levels were higher in RF-positive patients than in RF-negative patients (*p* = 0.02) (Table 2). There were no significant differences in posttreatment CXCL10 and CXCL13 levels between RF-positive and RF-negative patients (*p* = 0.57 and *p* = 0.72 respectively), and anti-CCP-positive and anti-CCP-negative patients (*p* = 0.09 and *p* = 0.21 respectively).

**Table 2:** Baseline chemokine levels in seropositive and seronegative RA patients. Values are presented as mean with standard deviation. *P* values were determined by Wilcoxon ranked sum test

	IgM RF+ (n = 16)	IgM RF- (n = 13)	*p* value
CXCL10 (pg/ml)	510.2 ± 465.2	400.4 ± 525.3	0.16
CXCL13 (pg/ml)	371.6 ± 649.0	40.3 ± 55.6	0.02*
CCL20 (pg/ml)	9.7 ± 10.3	23.8 ± 30.5	0.09
	Anti-CCP+ (n = 15)	Anti-CCP- (n = 14)	*p* value
CXCL10 (pg/ml)	557.3 ± 458.0	357.8 ± 512.9	0.02*
CXCL13 (pg/ml)	396.6 ± 663.8	37.2 ± 54.4	0.005*
CCL20 (pg/ml)	9.6 ± 9.9	22.9 ± 29.7	0.12

*IgG* immunoglobulin G, *RF* rheumatoid factor, *CXCL10* C-X-C motif chemokine 10, *CXCL13* C-X-C motif chemokine 13, *CCL20* C-C motif chemokine 20, *anti-CCP* anti-cyclic citrullinated peptide

*p < 0.05

### CXCL10 and CXCL13 levels decrease after TNF inhibitor therapy in responders

Posttreatment chemokine levels at 14 weeks after TNF inhibitor therapy were measured and compared to pretreatment (baseline) levels (Table 3). In responders, CXCL10, CXCL13, and CCL20 levels were decreased after TNF inhibitor therapy (*p* = 0.01, *p* = 0.0001, and *p* = 0.003 respectively). In nonresponders, CXCL13 levels did not change (*p* = 0.86) and CXCL10 and CCL20 levels were decreased (*p* = 0.04 and *p* = 0.047 respectively). The percentage change was compared between responders and nonresponders. CXCL13 levels were decreased by 62 % of baseline levels after TNF inhibitor therapy in responders, and increased by 6 % in nonresponders (*p* = 0.08). There was no difference in the percentage change of CXCL10 and CCL20 between responders and nonresponders.

**Table 3:** Pretreatment (baseline) and posttreatment chemokine levels in responders and nonresponders. *P* values were determined by analysis of variance (ANOVA) for repeated measures

	Responders		Nonresponders	
	mean ± SD	*p* value	mean ± SD	*p* value
CXCL10 (pg/ml)				
Pretreatment	605.5 ± 580.6	0.01*	283.2 ± 265.1	0.04*
Posttreatment	411.1 ± 458.3		218.4 ± 237.3	
CXCL13 (pg/ml)				
Pretreatment	382.7 ± 644.3	<0.0001*	26.7 ± 23.8	0.86
Posttreatment	90.6 ± 128.2		35.7 ± 46.8	
CCL20 (pg/ml)				
Pretreatment	13.6 ± 12.7	0.003*	19.0 ± 31.0	0.047*
Posttreatment	6.9 ± 8.9		7.2 ± 7.2	

*CXCL10* C-X-C motif chemokine 10, *CXCL13* C-X-C motif chemokine 13, *CCL20* C-C motif chemokine 20

*p < 0.05

### Baseline CXCL10 and CXCL13 levels predict response to TNF inhibitor therapy

RA patients were then classified into four groups (high CXCL10/high CXCL13, high CXCL10/low CXCL13, low CXCL10/highCXCL13, low CXCL10/low CXCL13) based on baseline CXCL10 and CXCL13 cutoffs defined by their median values (260 pg/ml and 50 pg/ml respectively), and their response to TNF inhibitor therapy was compared. Ten out of 12 patients in the high CXCL10/high CXCL13 group were responders, and nine out of 12 patients in the low CXCL10/low CXCL13 group were nonresponders.

**Fig. 2:**
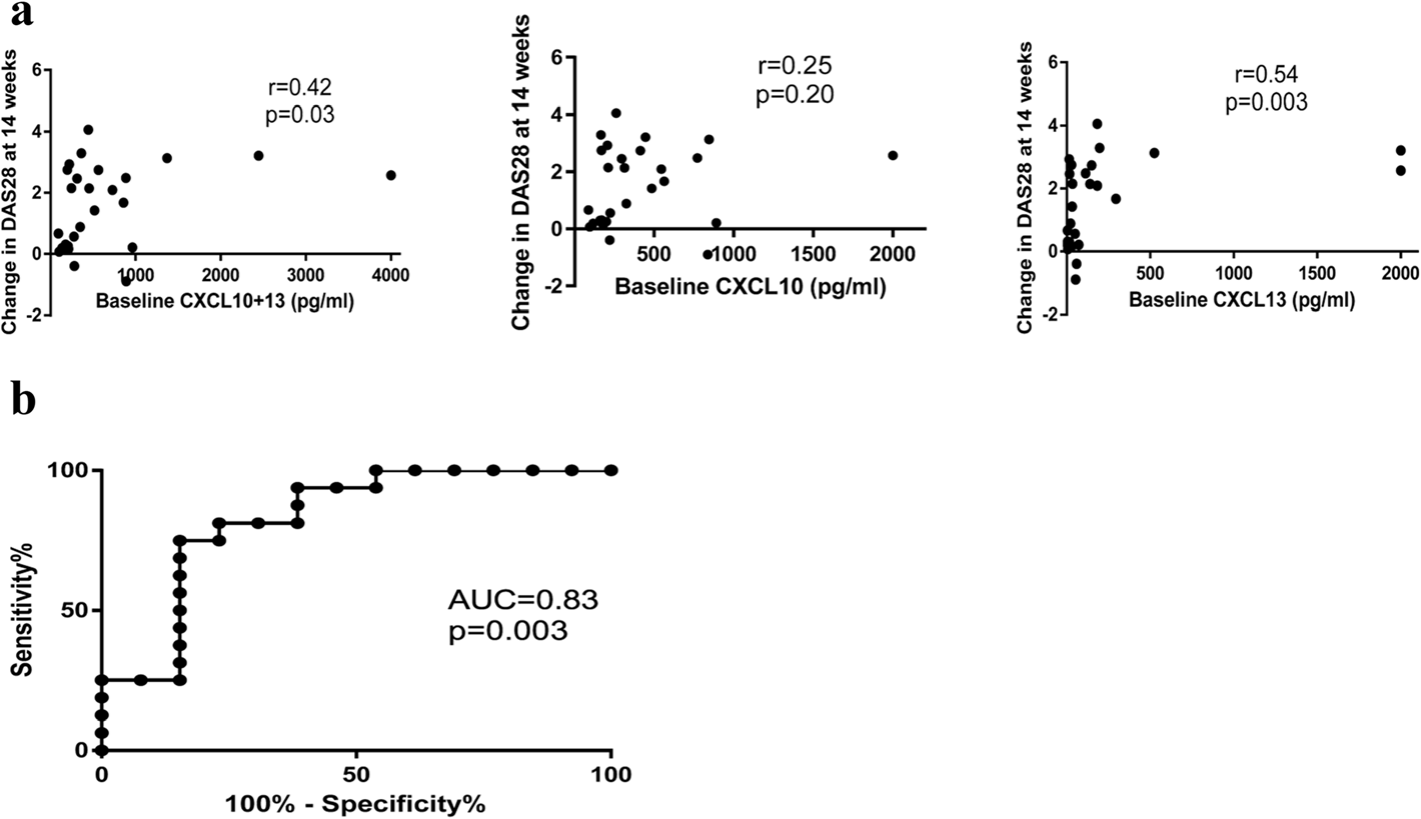
**a** Correlations between baseline CXCL10 + 13, CXCL10, CXCL13 and change in DAS28 at 14 weeks after TNF inhibitor therapy. Baseline CXCL10 + 13 (r = 0.42, *p* = 0.03) and CXCL13 (r = 0.54, *p* = 0.003) were correlated with change in DAS28 at 14 weeks. The associations between chemokine levels and change in DAS28 were assessed using Spearman correlation. **b** Predictive ability of CXCL10 + 13 for the response to TNF inhibitor therapy at 14 weeks. Area under the curve (AUC) in ROC curve analysis is 0.83

A chemokine score, CXCL10 + 13, was created by simply adding baseline CXCL10 and CXCL13 levels. There was a significant difference in baseline CXCL10 + 13 between responders and nonresponders (988 ± 1050 vs 310 ± 283 pg/ml, *p* = 0.006). Baseline CXCL10 + 13 and CXCL13 were correlated with changes in DAS28 at 14 weeks after TNF inhibitor therapy (r = 0.42, *p* = 0.03 and r = 0.54, *p* = 0.003 respectively), and CXCL10 levels were not correlated (r = 0.25, *p* = 0.20) (Fig. 2a). ROC curve analysis was performed to assess the predictive ability of CXCL10 + 13 for EULAR good or moderate response to TNF inhibitor therapy. CXCL10 + 13 showed significant predictive ability based on the area under the curve (AUC) of 0.83 (Fig. 2b).

## Discussion

This study demonstrates that baseline CXCL10 and CXCL13 levels are associated with favorable response to TNF inhibitor therapy in moderate to severe RA patients. On the other hand, CCL20 levels were relatively low in RA patients and there was no difference between responders and nonresponders. When analyzed separately based on the TNF inhibitors patients received, the results were similar, except that the difference for CXCL10 in patients treated with etanercept was not statistically significant, presumably due to the low subject number in this group. All the patients included in this study had inadequate response to methotrexate and had high disease activity measured by DAS28 when starting TNF inhibitor therapy.

CXCL10 is induced by type I and II IFNs [8, 9] as well as TNF [20], and has been evaluated as a surrogate biomarker to reflect the IFN signature upregulated in autoimmune rheumatic diseases including RA and systemic lupus erythematosus (SLE) [21, 22]. A previous study has shown that infliximab therapy leads to significant decrease in serum CXCL10 levels in RA patients [23], which is consistent with our study’s findings. Of note, RA patients with high baseline type I IFN activity have a good response to TNF inhibitors [4]. Additionally, it has been reported that type I IFN signature is induced by TNF in RA patients [24].

CXCR3-expressing Th1 lymphocytes and monocytes recruited by CXCL10 secrete TNF in RA [10, 11]. Furthermore, TNF induces type I IFN synthesis by macrophages and fibroblast-like synoviocytes, which in turn stimulates CXCL10 secretion in an autocrine manner and creates a positive feedback loop [25, 26]. In this regard, CXCL10 may be used as a marker associated with both TNF and type I IFN pathways, which are closely linked to each other in RA. This study provided additional evidence in support of the above notion.

CXCL13 is constitutively expressed by follicular dendritic cells and is involved in formation of secondary lymphoid tissues in RA [16]. Additionally, it is upregulated in a subset of T cells by T cell receptor engagement and by TNF or IL-6 [14, 15] as well as in macrophages [27]. CXCL13 is implicated in germinal center formation by B lymphocytes and follicular helper T lymphocytes (TFH), and likely in their recruitment into the inflamed synovial tissues in RA, where they may contribute to pathogenesis by generating local immune responses and antibody production. Immunoglobulin G (IgG)-containing immune complexes stimulate TNF production by monocytes via direct activation of Fc gamma receptor III (FcγRIII) [28]. It has been reported that CXCL13 was higher in seropositive RA patients than in seronegative RA patients [29, 30], and our study’s findings are consistent with these reports. CXCL13 is associated with severe RA with persistent ultrasonographic synovitis despite nonbiologic DMARDs therapy [31].

There have been two inconsistent reports on the roles of CXCL13 as a predictive marker for the response to TNF inhibitor in RA patients [12, 13]. In the first study, baseline CXCL13 levels in early RA patients were inversely correlated with disease activity at 12 months after DMARDs and adalimumab therapy [12], which is consistent with the findings of our study. In the second study, CXCL13 was associated with lower ACR50 response rate to adalimumab and higher ACR50 response rate to tocilizumab, an IL-6 receptor inhibitor, after 24 weeks of therapy [13]. The study was a post hoc analysis of ADalimumab ACTemrA (ADACTA) trial results where tocilizumab monotherapy and adalimumab monotherapy were compared [32]. ROC analysis in that study has shown CXCL13 has only modest predictive ability with an AUC of 0.6 [13]. As opposed to the ADACTA trial, all but one patient in our study were continued on baseline methotrexate and other DMARDs while taking TNF inhibitor. Furthermore, baseline CXCL13 and CXCL10 levels were highly correlated with each other in our study, and CXCL13 levels decreased after TNF inhibitor therapy only in responders. However, since CXCL13 was found to be a predictive biomarker for the response to tocilizumab [13], it would not be clinically helpful in choosing between TNF inhibitors and tocilizumab.

Response of RA patients to TNF inhibitor therapy could be associated with quality (i.e., subset) or quantity (i.e., severity) of synovial inflammation. CXCL10 and CXCL13 levels were not associated with disease activity in this study, and the present data are in favor of a qualitative rather than quantitative association between baseline CXCL10 and CXCL13 levels and the therapeutic response to TNF inhibitors. Therefore, we hypothesize that patients with high CXCL10 and high CXCL13 define a subset of RA whose inflammatory reactions are primarily driven by TNF and thus respond to TNF inhibitor therapy. The potential mechanistic interactions between CXCL10 and CXCL13 in RA remain to be determined in further studies. Additionally, it has been reported that C-C motif chemokine 19 (CCL19) predicts the clinical response to rituximab in RA [33], and we plan to further investigate this finding in future studies.

This study has several limitations. First, the study population is from a single tertiary care center and the results need to be confirmed in a larger population. There is furthermore a possibility that the data may have been biased by confounders, which may be better controlled in future studies by stricter inclusion and exclusion criteria and a study protocol. On the other hand, these data reflect findings from a real clinical practice setting. Another limitation is that this study did not include a control group of healthy individuals. Serum levels of CXCL10 and CXCL13 in RA patients are significantly higher than in healthy controls [34], and future studies will be needed to assess whether chemokine levels in the subsets of RA patients based on the clinical response to TNF inhibitor therapy are higher than the normal range. While some RA patients may take longer than 14 weeks to respond to TNF inhibitor therapy, many nonresponders in this study switched to a different agent after 14 weeks, preventing us from assessing later clinical responses.

## Conclusions

In conclusion, our results show that serum levels of CXCL10 and CXCL13 may be valuable predictive biomarkers of response to TNF inhibitor therapy. The decrease of CXCL10 and CXCL13 after treatment in responders is consistent with their biological significance in the disease process. Importantly, CXCL10 and CXCL13 levels are not correlated with disease activity or severity, but rather appear to be markers for a subset of RA patients whose inflammatory reactions are driven by TNF. Replication of these results in larger, controlled cohorts will be necessary to confirm their significance.

## Abbreviations

ACR: American College of Rheumatology
ADACTA: ADalimumab ACTemrA
ANOVA: analysis of variance
anti-CCP: anti-cyclic citrullinated peptide
AUC: area under the curve
CCL20: C-C motif chemokine 20
CCR6: C-C chemokine receptor type 6
CXCL10: C-X-C motif chemokine 10
CXCL13: C-X-C motif chemokine 13
CXCR3: C-X-C chemokine receptor type 3
CXCR5: C-X-C chemokine receptor type 3
DAS: disease activity score
DMARDS: disease-modifying antirheumatic drugs
ELISA: enzyme-linked immunosorbent assay
ESR: erythrocyte sedimentation rate
EULAR: European League Against Rheumatism
IFN: interferon
IL-1: interleukin 1
IL-17: interleukin 17
IL-6: interleukin 6
RA: rheumatoid arthritis
RF: rheumatoid factor
ROC: receiver operating characteristic
SLE: systemic lupus erythematosus
TFH: T follicular helper
Th1: T helper 1
Th17: T helper 17
TNF: tumor necrosis factor

## References

[1] Firestein GS (2003). Evolving concepts of rheumatoid arthritis. Nature.

[2] Klareskog L, Rönnelid J, Lundberg K, Padyukov L, Alfredsson L (2008). Immunity to citrullinated proteins in rheumatoid arthritis. Annu Rev Immunol.

[3] McInnes IB, Schett G (2011). The pathogenesis of rheumatoid arthritis. N Engl J Med.

[4] Mavragani CP, La DT, Stohl W, Crow MK (2010). Association of the response to tumor necrosis factor antagonists with plasma type I interferon activity and interferon-beta/alpha ratios in rheumatoid arthritis patients: a post hoc analysis of a predominantly Hispanic cohort. Arthritis Rheum.

[5] Liu C, Batliwalla F, Li W, Lee A, Roubenoff R, Beckman E (2008). Genome-wide association scan identifies candidate polymorphisms associated with differential response to anti-TNF treatment in rheumatoid arthritis. Mol Med.

[6] Thurlings RM, Boumans M, Tekstra J, van Roon JA, Vos K, van Westing DM (2010). Relationship between the type I interferon signature and the response to rituximab in rheumatoid arthritis patients. Arthritis Rheum.

[7] Raterman HG, Vosslamber S, de Ridder S, Nurmohamed MT, Lems WF, Boers M (2012). The interferon type I signature towards prediction of non-response to rituximab in rheumatoid arthritis patients. Arthritis Res Ther.

[8] Luster AD, Ravetch JV (1987). Biochemical characterization of a gamma interferon-inducible cytokine (IP-10). J Exp Med.

[9] Chiche L, Jourde-Chiche N, Whalen E, Presnell S, Gersuk V, Dang K (2014). Modular transcriptional repertoire analyses of adults with systemic lupus erythematosus reveal distinct type I and type II interferon signatures. Arthritis Rheumatol.

[10] Antonelli A, Ferri C, Ferrari SM, Colaci M, Fallahi P (2008). Immunopathogenesis of HCV-related endocrine manifestations in chronic hepatitis and mixed cryoglobulinemia. Autoimmun Rev.

[11] Lee EY, Lee ZH, Song YW (2013). The interaction between CXCL10 and cytokines in chronic inflammatory arthritis. Autoimmun Rev.

[12] Greisen SR, Schelde KK, Rasmussen TK, Kragstrup TW, Stengaard-Pedersen K, Hetland ML (2014). CXCL13 predicts disease activity in early rheumatoid arthritis and could be an indicator of the therapeutic ‘window of opportunity’. Arthritis Res Ther.

[13] Dennis G, Holweg CT, Kummerfeld SK, Choy DF, Setiadi AF, Hackney JA (2014). Synovial phenotypes in rheumatoid arthritis correlate with response to biologic therapeutics. Arthritis Res Ther.

[14] Manzo A, Vitolo B, Humby F, Caporali R, Jarrossay D, Dell'accio F (2008). Mature antigen-experienced T helper cells synthesize and secrete the B cell chemoattractant CXCL13 in the inflammatory environment of the rheumatoid joint. Arthritis Rheum.

[15] Kobayashi S, Murata K, Shibuya H, Morita M, Ishikawa M, Furu M (2013). A distinct human CD4+ T cell subset that secretes CXCL13 in rheumatoid synovium. Arthritis Rheum.

[16] Shi K, Hayashida K, Kaneko M, Hashimoto J, Tomita T, Lipsky PE (2001). Lymphoid chemokine B cell-attracting chemokine-1 (CXCL13) is expressed in germinal center of ectopic lymphoid follicles within the synovium of chronic arthritis patients. J Immunol.

[17] Alzabin S, Abraham SM, Taher TE, Palfreeman A, Hull D, McNamee K (2012). Incomplete response of inflammatory arthritis to TNFα blockade is associated with the Th17 pathway. Ann Rheum Dis.

[18] Chabaud M, Page G, Miossec P (2001). Enhancing effect of IL-1, IL-17, and TNF-alpha on macrophage inflammatory protein-3alpha production in rheumatoid arthritis: regulation by soluble receptors and Th2 cytokines. J Immunol.

[19] Zrioual S, Toh ML, Tournadre A, Zhou Y, Cazalis MA, Pachot A (2008). IL-17RA and IL-17RC receptors are essential for IL-17A-induced ELR+ CXC chemokine expression in synoviocytes and are overexpressed in rheumatoid blood. J Immunol.

[20] Ohmori Y, Wyner L, Narumi S, Armstrong D, Stoler M, Hamilton TA (1993). Tumor necrosis factor-alpha induces cell type and tissue-specific expression of chemoattractant cytokines in vivo. Am J Pathol.

[21] Karonitsch T, von Dalwigk K, Steiner CW, Blüml S, Steiner G, Kiener HP (2012). Interferon signals and monocytic sensitization of the interferon-γ signaling pathway in the peripheral blood of patients with rheumatoid arthritis. Arthritis Rheum.

[22] Rose T, Grützkau A, Hirseland H, Huscher D, Dähnrich C, Dzionek A (2013). IFNα and its response proteins, IP-10 and SIGLEC-1, are biomarkers of disease activity in systemic lupus erythematosus. Ann Rheum Dis.

[23] Eriksson C, Rantapää-Dahlqvist S, Sundqvist KG (2013). Changes in chemokines and their receptors in blood during treatment with the TNF inhibitor infliximab in patients with rheumatoid arthritis. Scand J Rheumatol.

[24] Gordon RA, Grigoriev G, Lee A, Kalliolias GD, Ivashkiv LB (2012). The interferon signature and STAT1 expression in rheumatoid arthritis synovial fluid macrophages are induced by tumor necrosis factor α and counter-regulated by the synovial fluid microenvironment. Arthritis Rheum.

[25] Yarilina A, Park-Min KH, Antoniv T, Hu X, Ivashkiv LB (2008). TNF activates an IRF1-dependent autocrine loop leading to sustained expression of chemokines and STAT1-dependent type I interferon-response genes. Nat Immunol.

[26] Rosengren S, Corr M, Firestein GS, Boyle DL (2012). The JAK inhibitor CP-690,550 (tofacitinib) inhibits TNF-induced chemokine expression in fibroblast-like synoviocytes: autocrine role of type I interferon. Ann Rheum Dis.

[27] Carlsen HS, Baekkevold ES, Morton HC, Haraldsen G, Brandtzaeg P (2004). Monocyte-like and mature macrophages produce CXCL13 (B cell-attracting chemokine 1) in inflammatory lesions with lymphoid neogenesis. Blood.

[28] Cooper DL, Martin SG, Robinson JI, Mackie SL, Charles CJ, Nam J (2012). FcγRIIIa expression on monocytes in rheumatoid arthritis: role in immune-complex stimulated TNF production and non-response to methotrexate therapy. PLoS One.

[29] Jones JD, Hamilton BJ, Challener GJ, de Brum-Fernandes AJ, Cossette P, Liang P (2014). Serum C-X-C motif chemokine 13 is elevated in early and established rheumatoid arthritis and correlates with rheumatoid factor levels. Arthritis Res Ther.

[30] Bugatti S, Manzo A, Vitolo B, Benaglio F, Binda E, Scarabelli M (2014). High expression levels of the B cell chemoattractant CXCL13 in rheumatoid synovium are a marker of severe disease. Rheumatology (Oxford).

[31] Bugatti S, Manzo A, Benaglio F, Klersy C, Vitolo B, Todoerti M (2012). Serum levels of CXCL13 are associated with ultrasonographic synovitis and predict power Doppler persistence in early rheumatoid arthritis treated with non-biological disease-modifying anti-rheumatic drugs. Arthritis Res Ther.

[32] Gabay C, Emery P, van Vollenhoven R, Dikranian A, Alten R, Pavelka K (2013). Tocilizumab monotherapy versus adalimumab monotherapy for treatment of rheumatoid arthritis (ADACTA): a randomised, double-blind, controlled phase 4 trial. Lancet.

[33] Sellam J, Rouanet S, Hendel-Chavez H, Miceli-Richard C, Combe B, Sibilia J (2013). CCL19, a B cell chemokine, is related to the decrease of blood memory B cells and predicts the clinical response to rituximab in patients with rheumatoid arthritis. Arthritis Rheum.

[34] Han JH, Suh CH, Jung JY, Nam JY, Kwon JE, Yim H (2015). Association of CXCL10 and CXCL13 levels with disease activity and cutaneous manifestation in active adult-onset Still's disease. Arthritis Res Ther.

